# The COVID – 19 pandemic influence on hospitalizations for ambulatory care-sensitive condition

**DOI:** 10.1093/eurpub/ckac131.307

**Published:** 2022-10-25

**Authors:** I Marasovic Šušnjara

**Affiliations:** 1 Public Health, Public Health Institute of Split-Dalmatia County, Split, Croatia; 2 University Department of Health Studies, University of Split, Split, Croatia

## Abstract

**Aim:**

To explore whether the COVID-19 pandemic influenced hospitalizations for ambulatory care-sensitive conditions (ACSCs) during the COVID-19 pandemic in Split-Dalmatia County, Croatia.

**Methods:**

We employed a cross-sectional comparative study using two different time periods, the prepandemic (March 2019 to February 2020) and the pandemic (March 2020 to February 2021) to explore possibilities of COVID-19 pandemic influence on hospitalizations for ambulatory care-sensitive conditions (ACSCs) during the COVID-19 pandemic in Split-Dalmatia County. The research used data from the national information system on hospitalizations from the Clinical Hospital Center Split, University Hospital Split. The ACSCs was classified in the categories of vaccine preventable, chronic and acute disease. The indicators were statistically analysed. The z-score test for two population proportions is used.

**Results:**

During prepandemic (March 2019 to February 2020) there were 48,289 hospitalizations, in the pandemic period (March 2020 to February 2021) there were 37,999 hospitalizations. The ACSCs hospitalizations made 6.4% in the prepandemic and 7.1% in the pandemic period. In the pandemic there was a significantly higher ACSCs hospitalizations compared to the prepandemic (z =-3.9348; p = 0.00008; p < 0), which was supported by a significant increase regarding ACSCs hospitalizations in the category of acute diseases, among women (z=-3.6614; p = 0.00026; p < 0 .05), in age groups 0-19 years (z=-4.0492; p < 0.00001; p < 0.05) and 20-64 years (z= -3.8818; p = 0 .0001; p < 0.05).

**Conclusions:**

The results of the study show that the COVID-19 pandemic contributed to the total number of hospitalizations as well as the hospitalization of the ACSC. One of the reasons for these changes was certainly the changed organization of the work of the entire health system due to the COVID-19 pandemic.

**Key messages:**

• The results of the study show that the COVID-19 pandemic contributed to the total number of hospitalizations as well as the hospitalization of the ACSC.

• One of the reasons for these changes was certainly the changed organization of the work of the entire health system due to the COVID-19 pandemic.

